# nRC: non-coding RNA Classifier based on structural features

**DOI:** 10.1186/s13040-017-0148-2

**Published:** 2017-08-01

**Authors:** Antonino Fiannaca, Massimo La Rosa, Laura La Paglia, Riccardo Rizzo, Alfonso Urso

**Affiliations:** ICAR-CNR, National Research Council of Italy, Via Ugo La Malfa, Palermo, 90146 Italy

**Keywords:** ncRNA, Classification, Structural features, Deep learning

## Abstract

**Motivation:**

Non-coding RNA (ncRNA) are small non-coding sequences involved in gene expression regulation of many biological processes and diseases. The recent discovery of a large set of different ncRNAs with biologically relevant roles has opened the way to develop methods able to discriminate between the different ncRNA classes. Moreover, the lack of knowledge about the complete mechanisms in regulative processes, together with the development of high-throughput technologies, has required the help of bioinformatics tools in addressing biologists and clinicians with a deeper comprehension of the functional roles of ncRNAs. In this work, we introduce a new ncRNA classification tool, nRC (non-coding RNA Classifier). Our approach is based on features extraction from the ncRNA secondary structure together with a supervised classification algorithm implementing a deep learning architecture based on convolutional neural networks.

**Results:**

We tested our approach for the classification of 13 different ncRNA classes. We obtained classification scores, using the most common statistical measures. In particular, we reach an accuracy and sensitivity score of about 74%.

**Conclusion:**

The proposed method outperforms other similar classification methods based on secondary structure features and machine learning algorithms, including the RNAcon tool that, to date, is the reference classifier. nRC tool is freely available as a docker image at https://hub.docker.com/r/tblab/nrc/. The source code of nRC tool is also available at https://github.com/IcarPA-TBlab/nrc.

## Background

During the last decade, research has shown a growing interest in non-coding RNA (ncRNA). They are small non-coding sequences with the potential to have a functional role in many biological processes and diseases [1] by acting through the regulation of gene expression [2–5]. Different classes of ncRNA have been identified, differing from each other by nucleotide sequence length, folding and function. The most well-known ncRNAs are structural RNA belonging to ribosomal RNA (rRNA) and transfer RNA (tRNA), both involved in translation events [6]. Another interesting class of ncRNA are microRNAs (miRNAs), 18–24 nucleotide long regulative RNA molecules [7–9]. They can behave as tumour suppressors or oncogenes depending on which target they act upon by altering the standard molecular mechanisms in which their targets are involved [10]. In particular, they interact with target genes through a direct binding to complementary sequences leading to either mRNA degradation or translational suppression [11]. The final result is the inhibition of the protein product. A miRNA can be considered as an oncogene if its amplification or overexpression down-regulates tumour suppressors or other genes involved in cell differentiation, thereby contributing to cancer formation by stimulating proliferation, angiogenesis, and invasion; whereas the ncRNA molecule will be considered as a tumor-suppressor if it will cause a decrease in oncogene expression [12].

Other ncRNA classes are small nuclear RNAs (snRNA), long non-coding RNAs (lncRNA), silencing RNA (siRNA), riboswitches and internal ribosome entry sites (IRES) [13]. The small nucleolar RNA (snoRNA) molecules, belonging to the snRNA class, participate to post-transcriptional modifications of rRNA, together with small nucleolar ribonucleoproteins (snoRNPs) with whom they are complexed. Dong and colleagues [14] reported a disruption of these RNA molecules in different conditions and cancer diseases [14, 15], they also identified snoRNA U50 as an important factor in the development and/or progression of breast cancer. The lncRNAs are ncRNA longer than 200 nucleotides. Recent works evidence a dysregulated expression pattern of lncRNAs in cancer samples that may be used as independent predictors of patient outcomes [16, 17]. Riboswitches are another class of ncRNA. They are structured non-coding RNA domains that selectively bind metabolites and control gene expression. They can act without the support of proteins, which strengthens the hypothesis of their important role in the regulatory machine [18].

Because of the large number and functions of different ncRNA, their proper identification and classification are a new challenging bioinformatics scenario. Indeed, considering the low percentage of the “discovered ncRNAome” and the lack of knowledge about these non-coding molecules, their classification could help biologists and clinicians in understanding the molecular mechanisms of this regulatory machine. This also implies a need to re-state the principles of basic therapeutic strategies.

The aim of the first works about ncRNA classification was to discriminate between coding and non-coding sequences. To this purpose some bioinformatics tools employ support vector machine (SVM) models [19]: CONC and CPC are prediction tools based on SVM that classify transcripts according to features belonging to coding products [20, 21]. Another interesting classification method, proposed by Lertampaiporn and colleagues [22], uses a hybrid Random Forest (RF) algorithm combined with a logistic-regression model that realises a feature-based discrimination among various ncRNAs. The recent discovery of a “Pandora box” full of a multitude of different biologically functional ncRNA, opened the way to develop resources able to discriminate the different classes of ncRNAs. Various approaches have been applied such as RNA-CODE [23], based on the alignment of short reads, or others based on multi-feature extraction and full-sequence analysis such as RNAcon [24] and GraPPLe [25]. These last methods, in particular, use graph properties (both local and global) of predicted secondary RNA structures together with machine learning algorithms. Their main feature is to identify and extract graph properties that can reflect the functional information of different classes of RNAs. To the best of our knowledge, the RNAcon algorithm currently represents the state-of-the-art classifier of ncRNA classes based on structural features and machine learning techniques. RNAcon considers 20 graph features obtained from the predicted RNA secondary structure and adopts an RF classifier [26].

In this paper, we present nRC (non-coding RNA Classifier), a novel method for the classification of ncRNA sequences belonging to different classes. Our approach uses the structural features extracted from ncRNA secondary structure, rather than the primary structure since it has been demonstrated that the structure of ncRNAs can provide relevant information about their biological functions and therefore their class type [27]. Moreover, we adopted a supervised classification algorithm implementing a deep learning (DL) architecture based on convolutional neural networks (CNNs) [28]. DL represents a successful paradigm for big data analysis, giving a relevant contribution to several fields of medicine and bioinformatics [29]. For instance, the use of DL architectures for the prediction of genomic sequences allows improving the performance of the other standard machine learning methods [30, 31].

In particular, CNNs have been successfully adopted for image classification [32] because they can extract significant features from images at different abstraction levels. Recently, CNNs have also been applied to DNA sequence classification [33] with good results, due to their capability to extract meaningful features even from sequences of symbols. The combination of both structural features and a DL architecture allows us to reach classification scores that outperform other similar classification methods based on secondary structure features and machine learning algorithms like the random forest (RF) [26] and naive Bayes (NB) [34] classifiers.

## Methods

### Proposed method

**Fig. 1:**
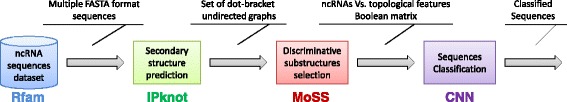
Pipeline of the proposed ncRNA sequence classification tool. Starting from a dataset of known ncRNA sequences, we exploit IPknot, MoSS and CNN algorithms in order to (1) predict ncRNA secondary structure, (2) select discriminative features and (3) accurately classify ncRNA classes. Texts over the arrows show the type or data format produced by the processing steps (*square blocks*) or the data source (*cylinder block*)

In this section, we introduce the proposed approach for the classification of ncRNA sequences. We classify ncRNA sequences by exploiting a set of discriminative substructures extracted from RNA secondary structures. Starting from a dataset composed of ncRNA fasta sequences belonging to different non-coding classes, we first predict the secondary structure of each sequence (Fig. 1). Then, we identify as features all the discriminative frequent sub-structures extracted from predicted ncRNA secondary structures. Finally, a supervised classification algorithm is trained using as input a ncRNA sequence vs. sub-structures boolean matrix. Each step of the proposed approach, corresponding to a box in Fig. 1, is detailed in the next subsections.

### ncRNA training dataset

**Fig. 2:**
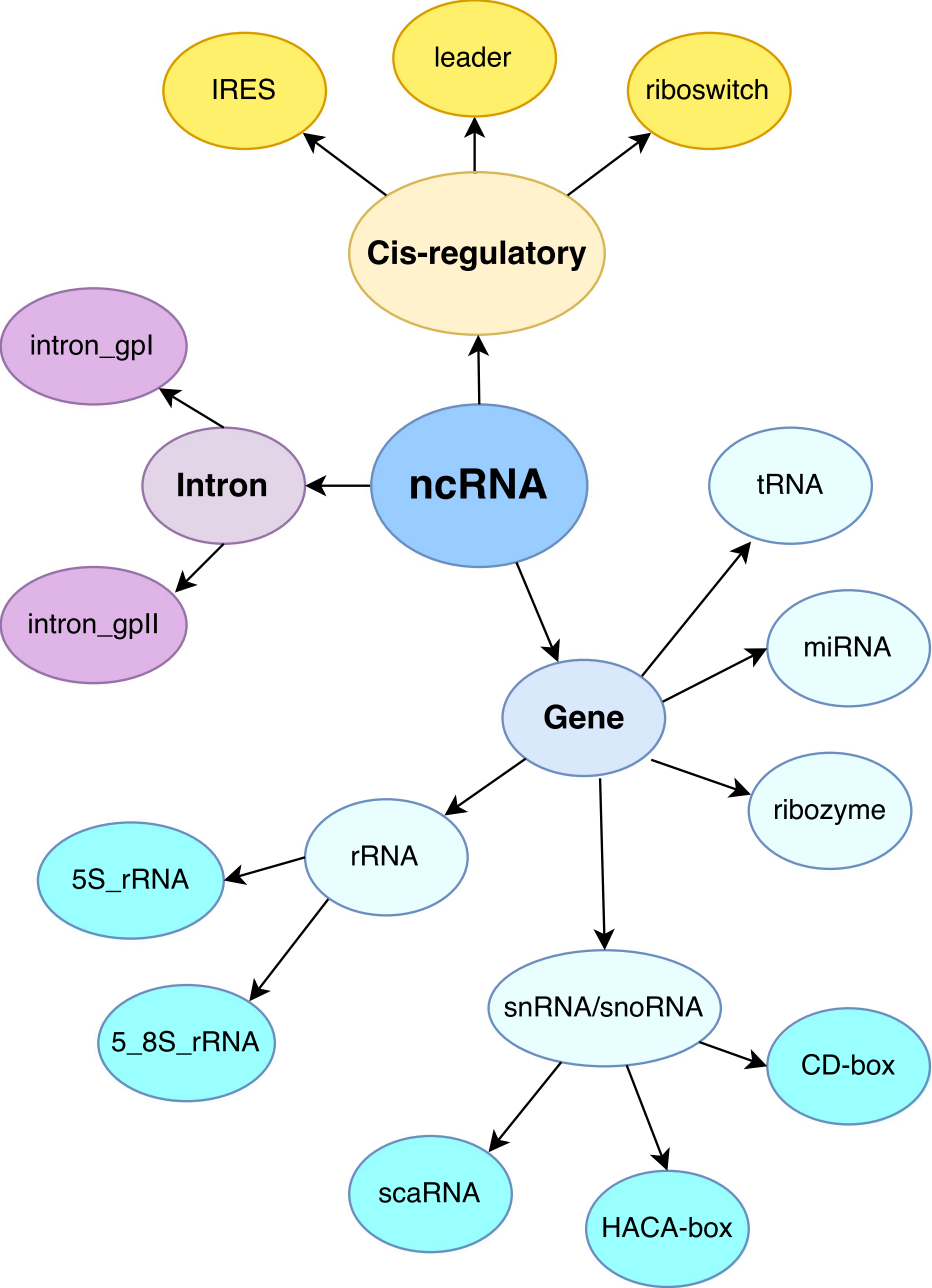
Hierarchical tree for ncRNA sequences dataset, according to the Rfam database. In this study, we selected 13 ncRNA classes, represented as the leaves of a hierarchical tree

To create a consistent and statistically meaningful ncRNA dataset, we followed the approach proposed by Panwar et al. [24], and by Childs et al. [25]. Similar to those studies, we downloaded the ncRNA sequences from the latest version of the Rfam database, release 12 [35]. The Rfam repository represents one of the most complete collections of manually curated RNA sequences, including sequence alignments, annotation and consensus secondary structures. We selected the following 13 ncRNA classes: miRNA, 5S rRNA, 5.8S rRNA, ribozymes, CD-box, HACA-box, scaRNA, tRNA, Intron gpI, Intron gpII, IRES, leader, riboswitch. As will be further explained below, we chose these classes to allow a comparison as fair as possible with RNAcon tool. According to Rfam hierarchical organisation among the selected ncRNA classes (Fig. 2), 5S rRNA and 5.8S rRNA belong to rRNA class; CD-box, HACA-box and scaRNA belong to snRNA/snoRNA class; Intron gpI and Intron gpII belong to Intron class. Generally speaking, the leaves of the hierarchical tree represent ncRNA classes used in this study. According to Rfam database, there are three main functional categories for non-coding RNA sequences, i.e. gene, intron or cis-regulatory element. Considering those ncRNA classes, we built a dataset composed of 20% non-redundant sequences obtained by using the CD-HIT tool [36], as done by Panwar et al. [24]. Finally, to create a balanced dataset, we randomly selected 500 sequences for each ncRNA class, except IRES for which there are only 320 available sequences, to obtain 6320 ncRNA sequences.

### ncRNA secondary structure prediction

**Fig. 3:**
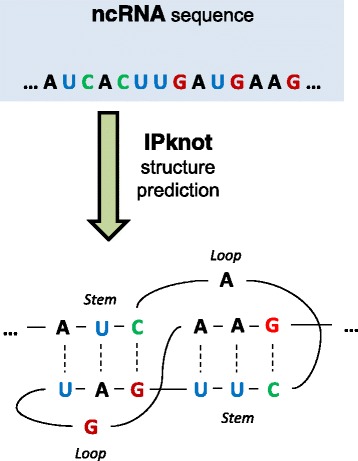
ncRNA secondary structure prediction. At the *top* of the figure is an ncRNA sequence, whereas the corresponding pseudoknot structure predicted by the IPknot tool is shown *underneath*

Since the ncRNA dataset reports sequences in fasta format, it does not contain information about the secondary structure of non-coding RNA sequences. As aforementioned in the previous section, the secondary molecular structures can provide a major key for elucidating the potential functions of RNAs and, consequently, could help us to predict if a ncRNA sequence belongs to the same class. For this reason, just as the RNAcon approach, we choose to exploit the IPknot tool [37] for predicting the secondary structure of ncRNA. This tool takes into account all the most important topologies in RNA secondary structures and can provide good predictions in terms of both speed and accuracy with respect to other RNA structure prediction methods [37]. To the best of our knowledge, IPknot is one of the best pseudoknot-free secondary structure prediction tools, since it uses less memory and runs much faster than the other tools, without loss of accuracy [38]. In our study, the most of ncRNA sequences, such as 5S rRNA, tRNA and miRNA, are pseudoknot-free [39]. Figure 3 shows how IPknot can predict a complex secondary structure. As a result, this tool produces a dot-parenthesis format file (representing a graph) for each input sequence.

### Discriminative sub-structure selection

Each dot-parenthesis format file prepared in the previous step can be read as an undirected labelled graph representing the RNA sequence, in which vertices are nucleotides and edges are bonds between two nucleotides. As mentioned before, we have 6320 graphs belonging to 13 ncRNA classes. Of course, we can reasonably suppose there is a sort of similarity among the sequences (graphs) that belong to a particular class. Our hypothesis is that frequent sub-structures (sub-graphs) can act as local features for describing ncRNA sequences because they are probably correlated with the molecular function and, thus, they can be used to identify classes of similar non-coding RNAs.

In this context, the selection of molecular sub-structures can be solved in terms of frequent sub-graphs having a certain minimum “support” in a given set of graphs, where the term support identifies the number of graphs containing a sub-graph. To find these sub-graphs, we adopted the molecular substructure miner (MoSS) algorithm [40], which implements a depth-first search strategy. The support expressed as a percentage value is the MoSS parameter that specifies the minimum frequency which a sub-structure must occur to be reported. In any case, since the search of frequent sub-graphs in a set of graphs can produce a very large number of features, the advanced pruning techniques implemented in the MoSS algorithm allows us only to obtain closed frequent sub-graphs. A sub-graph is closed only if its support (i.e., the number of graphs that contain this sub-graph) is higher than the support of all the search tree super-graphs containing this sub-graph. Also, the MoSS algorithm lets the user set the *m* minimum and the *n* maximum size the sub-structures must have to be taken into account. In the field of molecular compounds, a similar approach was applied to find potential candidates in drug discovery processes [41, 42].

**Fig. 4:**
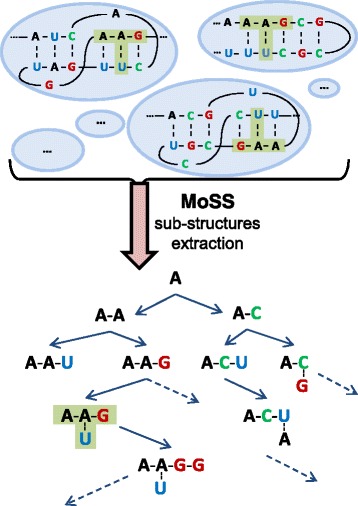
An example of discriminative sub-structures selection implemented by the MoSS algorithm. The *bottom* of the figure shows a decision tree, where nodes are sub-structures into input sequences. The *green* ’T’ shape highlights a sub-structure contained in both predicted structure and decision tree. The feature model proposed in nRC tool is given by the set of discriminative sub-structures

As an example, Fig. 4 shows a search tree (starting from an adenine nucleotide as a seed) created by the MoSS algorithm, when the input is a list of graphs (such as those reported in the top of the figure). In this figure, a sub-graph, i.e. a node of the search tree, is highlighted with a green ’T’ shape area in both predicted secondary structure and the search tree. That means that this sub-graph is a support for the ncRNA sequence at the top of the figure; if it is also a support for a certain user-determined percentage of input sequences and its super-graph has a lower support, it can be considered as a feature of a ncRNA dataset.

Outcomes of the MoSS algorithm are both the list of closed frequent sub-graphs and, for each graph, the list of its closed sub-graphs. Given *g* graphs (ncRNA sequences) and *s* sub-graphs (frequent sub-structures), it is possible to define a Boolean matrix *A*(*g*,*s*) where the element (*i*, *j*) is set to 1 when sub-structure *j* is contained in an ncRNA sequence *i*.

### Classification with deep learning architecture

A machine learning classifier requires a hand-crafted feature selection task to obtain the best representation of the input patterns; this step is crucial for the performances of the classifier. Automatic feature selection is one of the key results of the so-called deep learning neural networks [28, 43, 44]. Le Cun and colleagues demonstrated that feature selection could be obtained from neural network training [32]. The proposed model, called convolutional neural network, was constituted by a set of layers based on convolutional filters and average pooling layers, followed by a multi-layer perceptron. Nowadays CNN networks are often used for image classification, in these applications the first layers of the network are trained to recognise features constituted by edges or colour details that are assembled to create more sophisticated features used as image descriptors [28]. These image descriptors represent the input of the last, fully connected layers of the network, that implement the classifier.

**Fig. 5:**
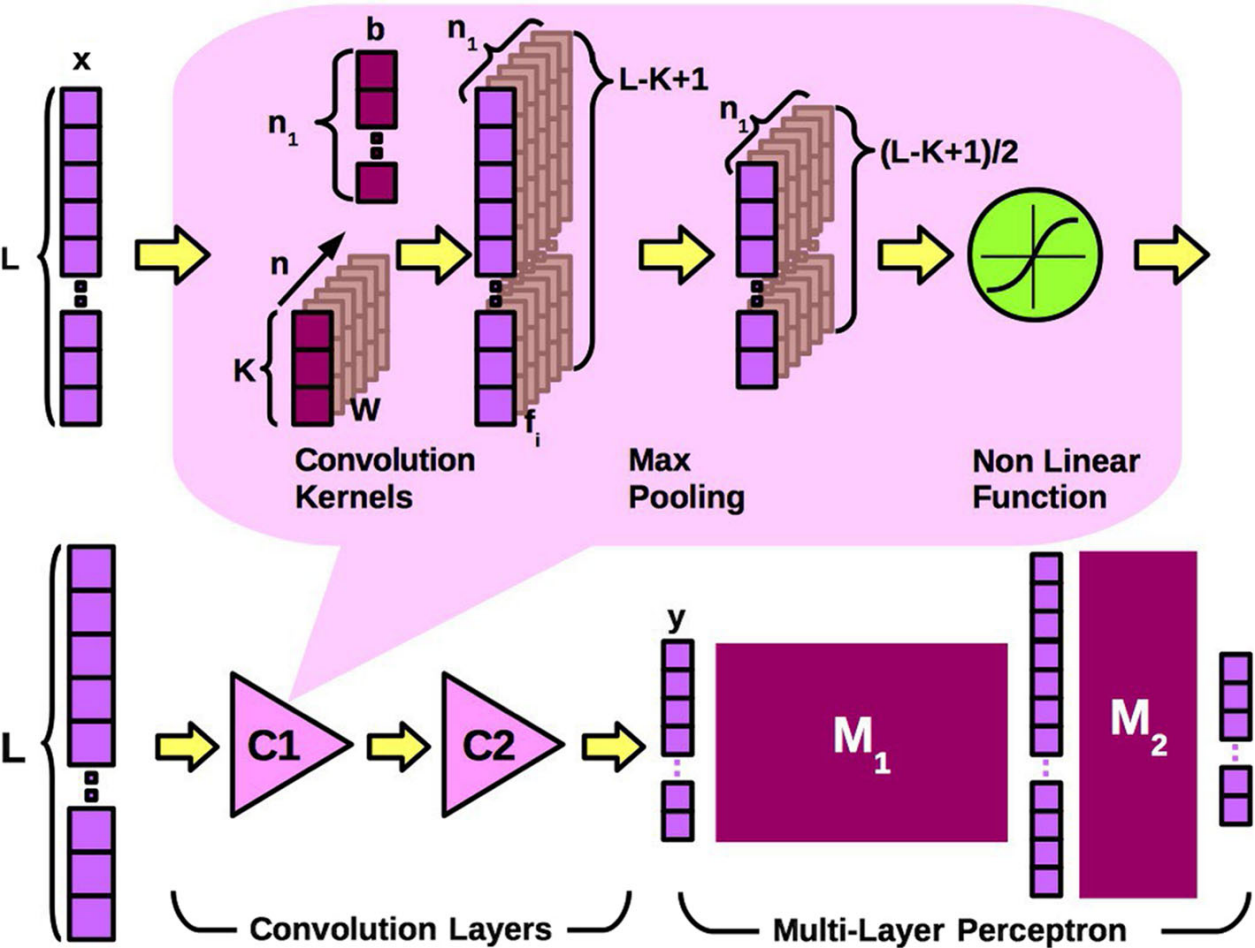
A representation of the convolutional neural network used in our work. The *lower* part is a representation of the network layers; the *upper* part is a representation of the operation in a convolutional layer with kernels (*dark purple*) and output vectors (*light purple*)

In the nRC system, each position of the vector obtained by the MoSS subsystem indicates the presence or absence of a structural configuration (Fig. 4). Even if the input vectors are binary and the vector components are not in a particular order, bit configurations can still be used as useful features and assembled to build new, more sophisticated patterns that a CNN can exploit. The neural network used in the nRC system is made by two convolutional layers, C1 and C2, followed by two fully connected layers (Fig. 5). The first convolutional layer C1 of the network learns to recognise features constituted by *n*_1_ groups of these binary values. The dimension of the convolutional kernels or filters in this first layer should be enough to capture interesting patterns but is upper limited by the computational time. In this work kernels from 3 to 8 were tried and *k*=5 was used because represents a compromise between length and computational load. The kernels are floating point vectors adjusted during training phase by the learning algorithm. Considering that the input vectors are binary, then an upper limit for the number of kernels *n*_1_ is due to the total number of configurations that can be obtained with *k* bits. If *k*=5, then the kernels can be 31 at most (excluding the configuration with all zeros values). To maintain a manageable training time we choose to use a *n*_1_=10 kernel for the first stage. The second convolutional layer of the network has kernels of the same dimension (*k*=4) and *n*_2_=20.

A CNN, like the one used in this work, is usually considered “deep” if compared with the commonly used multilayer perceptrons that usually have three layers (input, hidden, and output). If the input pattern is a vector *x*∈ℜ^*L*^ and the layer C1 uses a set of *n*_1_ kernels *w* of dimension *k* (*w*∈ℜ^*k*^), the convolution output will be a set of *n*_1_ vectors *f*_*i*_∈ℜ^*L*−*k*+1^.

1 $$ f_{i}=w_{i} \star x + b_{i} \ \ i=1, 2, \dots, n_{1}  $$

where ⋆ indicates the convolution operator and *b*_*i*_ is an offset parameter. A logistic function is a non-linear function applied to the output in the proposed application. The output vectors are reduced using the max-pooling operation with a pool of dimension two so that the resulting output vectors will be (*L*−*K*+1)/2. The max pooling layer compresses the input representation from *C*_1_ layer and allows to obtain a more dense representation of the input data. The C2 layer has the same structure but operates with a multi-dimensional input, the output of the C2 layer is constituted by a set of *n*_2_ vectors *f*_*j*_ *j*=1,2,…,*n*_2_ given by:

2 $$ f_{j}=\sum_{l} w_{j,l} \star x_{l} + b_{j} \ \ j=1, 2, \dots, n_{2}.  $$

The *f*_*j*_ vectors are rearranged in a single vector *y*, containing the features extracted from the input pattern. This vector is the input to a fully connected multi-layer perceptron, with only one hidden layer. The whole network is trained using the stochastic gradient descent algorithm and is implemented in Python using the Theano framework [45, 46].

### Implementation details

According to the introduced pipeline, we integrated the following publicly available algorithms: Ipknot (release 0.0.2), MoSS (release 2.13) and Theano (release 0.8.2). The docker image is based on the operating system Linux Centos (release 7.2.1511). Java (release 1.8.0) and Python (release 2.7.5) were the languages used to implement the nRC tool.

## Results

In this section, we presented the classification results obtained by our classification pipeline. We performed two kinds of experiments: in the first one, we tested nRC tool using a ten-fold cross-validation scheme to find the best configuration in terms of a number of structural features and parameters of the CNN model. In the second one, we validated the best models obtained during the testing phase by considering an independent dataset, downloaded from Rfam database, and consisting of 2600 sequences, not used in the training phase, belonging to the same 13 ncRNA classes as the training dataset. That validation procedure assured us that there is not overfitting with regards to both feature extraction and the learning of the CNN. We introduced both the number of local features and the statistical measures used for testing procedures. Then, since we want to demonstrate that in the proposed pipeline a deep learning architecture can outperform standard classification techniques, we compared the CNN algorithm with 4 of the most knowns supervised classifiers. Moreover, to test our method against RNAcon tool, the state-of-the-art technique for classification of ncRNA sequences, we introduced an independent validation dataset. Finally, we discussed the obtained results.

### Testing procedures

To evaluate our method for classification of ncRNA sequences, we developed a testing procedure considering different values of the minimum and maximum size of the frequent subgraph fragments extracted by the MoSS tool. Each configuration of those parameters gave, in fact, a different number of structural features used by the classification algorithms. We considered five different configurations, with sub-fragment sizes ranging from two to six, because we are interested in considering local features. The chosen size produced a different number of input features: in particular, we obtained only a few features (about 250) up to many features (about 6000) with regards to the number of sequences in our dataset, i.e. 6320. The min and max size of the MoSS sub-graphs are from 2/4 to 3/6 and the corresponding number of features range from 250 to 6483 (Table 1).

**Table 1:** Number of input features related to the min (m) and max (n) size of the frequent sub-structures extracted by the MoSS algorithm

MoSS parameters		Number of input features
m/n	Dataset support (%)	
2/4	10	250
4/5	10	1258
3/5	10	1298
4/6	10	6443
3/6	10	6483

Classification performances have been computed using a ten-fold cross-validation procedure in terms of accuracy, precision, sensitivity, specificity, F-score and MCC. These statistical measures are defined in Table 2.

**Table 2:** Statistical measures and their formulas used for evaluating the classifiers

Statistical measure	Formula
Accuracy	$\frac {TP + TN}{TP + TN + FP+ FN}$
Sensitivity	$\frac {TP}{TP+FP}$
Specificity	$\frac {TN}{TN + FP}$
Precision	$\frac {TP}{TP + FP}$
F-score	$\frac {2*TP}{2*TP + FP + FN}$
MCC	$\frac {TP*TN - FP*FN}{\sqrt {(TP + FP)(TP+FN)(TN+FP)(TN+FN)}}$

TP are true positives, TN are true negatives, FP are false positives, FN are false negatives

### Comparison among CNN and other machine learning algorithms

**Fig. 6:**
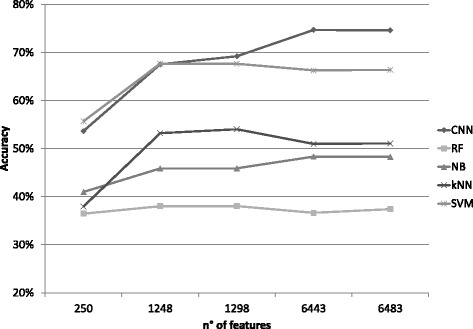
Accuracy scores. Scores at varying the number of input features, for our deep learning approach and the other four considered classifiers. The results are averaged over cross-validation experiments

Our proposed classifier based on DL architecture has been compared with four state-of-the-art feature-based algorithms: NB [34], RF [26], k nearest neighbour (kNN) [47] and support vector machine (SVM) [19]. All these algorithms were run using the Weka 3.6.13 platform [48]. As done for the CNN parameters, the one introduced in “Classification with deep learning architecture” section, we made several trials with different parameter values to establish, for each classification algorithm, the configuration that gave the best performances in terms of evaluation criteria. In detail, on the default algorithm configurations in the Weka platform, we set the following parameters: NB with kernel estimator option, RF with 100 trees and seed = 10, kNN with *K* = 3, SVM with gamma = 0.01 and cost = 10. As regards the CNN, the kernel size is *k*=5 for both first and second layer; the pool size is 2 for both layers; the number of kernels is *n*_1_=10 for the first layer and *n*_2_=20 for the second layer. In the fully connected layer, the number of hidden units (columns of M_1_ and rows of M_2_) was 500. We did the first comparison to consider how the accuracy scores change according to the five different numbers of the input features (see Table 1). Our DL approach reaches the highest score of about 74.7*%* when considering the 6443 features (Fig. 6). The second best classifier is the SVM, with a max accuracy score of about 67.36*%* when considering 1258 features. The remaining three classifiers did not provide satisfying results.

Considering the results obtained with all the classifier algorithms and all the performances indexes, we found that the CNN network results have the lowest standard deviation for all the measures (Table 3). Moreover, the value of all the performance indices increases with the number of input features (Table 4).

**Table 3:** Comparison among five classification algorithms (Alg.) in terms of percentage scores (%) and standard deviations (*σ*) of five statistical measures

Alg.	Accuracy			Sensitivity			Specificity			Precision			F-score			MCC	
	%	*σ*		%	*σ*		%	*σ*		%	*σ*		%	*σ*		%	*σ*
CNN	74.69	0.013		74.13	0.010		97.89	0.001		74.09	0.010		74.11	0.010		72.59	0.10
RF	56.60	1.544		56.50	1.539		96.41	0.001		55.69	0.019		56.10	0.037		52.4	0,16
NB	47.31	1.837		48.98	1.844		95.61	0.001		46.80	0.019		47.87	0.037		43.4	0.16
kNN	54.70	1.609		59.17	1.783		96.28	0.001		54.26	0.018		56.61	0.035		51.2	0.15
SVM	67.36	1.991		67.47	1.855		97.11	0.001		73.92	0.673		67.76	0.019		63.1	0.16

For each algorithm, the number of input features providing the best scores has been considered. The results are averaged over cross-validation experiments

**Table 4:** Statistical measures for CNN algorithm classifications

Number of	CNN statistical measures (%)					
features	Accuracy	Sensitivity	Specificity	Precision	F-score	MCC
250	53.66	53.18	96.14	52.87	53.03	49.81
1258	67.55	68.58	97.44	68.30	68.44	64.85
1298	69.22	68.61	97.43	68.59	68.60	66.65
6443	74.69	74.13	97.89	74.09	74.11	72.59
6483	74.60	74.01	97.88	73.97	73.99	72.48

According to the MoSS parameter configurations, here we report five sets of features. The results are averaged over cross-validation experiments

During the evaluation procedure, we also compared the execution time among the classification algorithms used in this study. As regards the training phase, the CNN algorithm is significantly more time consuming with respect to the other algorithms, a it is based on DL architecture. Fortunately, in the most case, the classification model is trained only once, so that users can exploit it for classifying new sequences. Conversely, as regards the testing phase, i.e. the classification of new sequences, the CNN is the second fastest algorithms just behind the RF. We report the average execution time taken to test classification models in Table 5. All experiments were carried out on a Windows 10 PC, with Intel i7 2.8 GHz CPU and 8 GB RAM.

**Table 5:** Comparison among average execution times of classification models, during the ten-fold cross-validation procedure

Algorithm	Execution time (seconds)
CNN	17.33
NB	25.97
KNN	29.36
RF	2.93
SVM	21.46

### Validation procedure

**Fig. 7:**
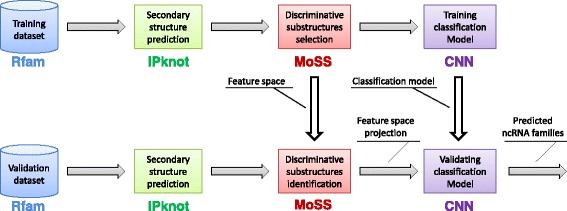
Validation pipeline. Through the training process (on the *top* of the figure), we obtain both feature space and classification model for a training dataset. Starting from an independent validation dataset, we can validate (on the *bottom* of the figure) the proposed algorithm, by projecting discriminative substructures belonging to ncRNA secondary structures into the feature space created during the training phase and then predict ncRNA classes using previous learned classification model

To further validate our proposed method we performed another classification experiment using an independent dataset whose elements have never been seen by the classifier during the learning phase. We downloaded the validation dataset from Rfam database and is composed of 2600 sequences belonging to the same 13 ncRNA classes as in the original dataset (200 sequences per class). To be more precise, we wanted to demonstrate that both the feature space of size 6443 and the CNN model, learned with the whole training dataset, can generalise the ncRNA class predictions, thus avoiding overfitting. To do that, we first predicted the secondary structure of validation sequences through IPknot, then we represented the sequences of the validation dataset in the same feature space created during the training phase (Fig. 7); finally we evaluated the best CNN model (see Table 4, fourth row) trained with the whole training dataset predicting the ncRNA classes of the validation dataset. The classification results confirm the robustness of the nRC tool with unknown data (Table 6).

**Table 6:** Statistical measures for nRC classification during the validation procedure

Number of features	CNN statistical measures					
	Accuracy	Sensitivity	Specificity	Precision	F-score	MCC
6443	81.81%	81.81%	98.48%	81.50%	81.66%	80.29%

It has been used an independent dataset composed of 2600 sequences belonging to 13 ncRNA classes

### Comparison between nRC and the RNAcon tool

**Fig. 8:**
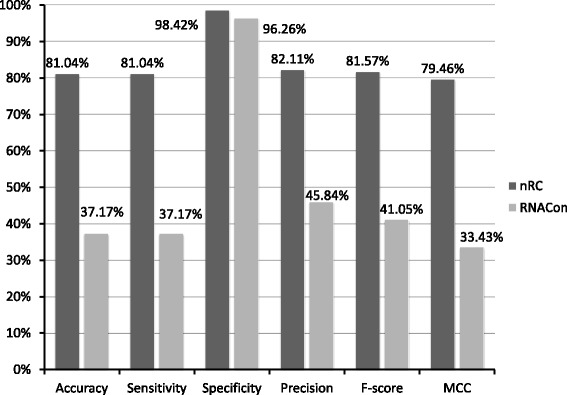
Comparison between the classification scores obtained by our approach and the RNAcon tool. The validation set is composed of 2400 sequences belonging to 12 classes

As explained at the end of the “Background” section, the RNAcon tool is the reference classifier of ncRNA sequences that consider structural features and machine learning algorithms. In particular, RNAcon extracts 20 local and global graph properties from the ncRNA predicted secondary structures, and it makes classification using the RF algorithm. Because our proposed method also considers structural features, the frequent sub-graphs, a machine learning classifier, i.e. the DL convolutional network, we made a direct comparison of our results with the ones provided by RNAcon. We used the RNAcon web service available at http://crdd.osdd.net/raghava/rnacon/, and we made the comparison considering the validation dataset because it represents an independent dataset for both tools. In particular, we removed from the validation dataset the sequences belonging to the scaRNA class, obtaining this way a set of 2400 sequences, because they are not present in the training dataset of RNACon. Our method outperforms RNAcon, doubling its performances according to accuracy and sensitivity scores when 6443 input features are considered (Fig. 8).

## Discussion

**Fig. 9:**
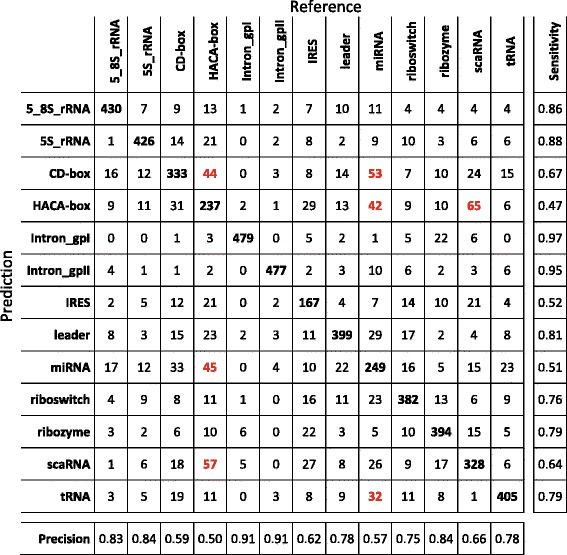
Confusion matrix related to the predictions of nRC tool over the training dataset (6320 sequences). It has been obtained putting together the single confusion matrices produced at the end of each fold during the testing procedure. Classification of Intron gpI and Intron gpII classes gave the best result; classification of IRES, miRNA and HACA-box classes gave the worst results. In *red* we highlighted some situations, some of which discussed in Discussion section, that will need further investigation

Because it has been proved that structural properties of the secondary structure of RNA molecules can provide specific information of the biological function of different ncRNA classes [27], we presented a classifier that works on a feature set representing frequent fragments of the RNA molecular structure. That representation, coupled with a classifier based on a DL architecture, allowed us to obtain the best scores when compared to other machine learning algorithms and the RNAcon tool. To analyse in detail the performances of our method, we produced the confusion matrix (Fig. 9), so that it is possible to inspect which ncRNA classes our approach better predicted. That confusion matrix has been obtained putting together the single confusion matrices produced at the end of each fold during the testing procedure. For example, we noticed that Intron_gpI and Intron_gpII classes are predicted with a sensitivity score of about 95%, whereas miRNA, IRES and HACA-box classes reached sensitivity and precision score of about 50%. We highlighted in red some situations that will need further investigation in the future. For example, the most misclassified miRNAs (9%) are predicted as HACA-box, correspondingly, 8.4% of HACA-box are predicted as miRNA. The same situation happened to the scaRNA class, with 11.4% misclassified as HACA-box, which in turn is predicted as scaRNA in 13% of cases.

As evidence, there is a misrepresentation of some ncRNA classes. As for the CD-box and HACA-box, both classes belong to the same main class group, i.e. they all are snoRNA (see Fig. 2). Even though they have a different global secondary structure, they could share local sub-structures, in fact, they are both involved in the chemical modification of the RNA classes rRNA, tRNA and snRNA after transcription, hypothesising a common link between their function and their structural sub-features. In particular, CD-box RNAs guide methylation events and HACA-box RNAs guide pseudouridylation of the RNA target [49]. Another RNA class belonging to snoRNA class is scaRNAs. The scaRNAs are involved in the modification of RNA polymerase II transcribed spliceosomal RNAs, and they are also defined as composite HACA- and CD-box RNAs, because their conserved domains are the typical motifs of both HACA-box and CD-box [50]. Moreover, similar sub-structures could be found in both miRNAs and some snoRNA, since recent reports have indicated that, despite the differences in size and secondary structure, a human snoRNA and a protozoan snoRNA are associated with Argonautes, processed into small RNAs, and can function as miRNAs [51, 52].

**Fig. 10:**
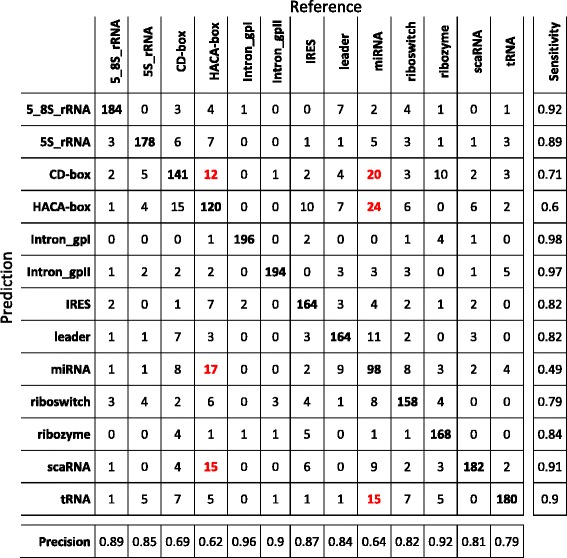
Confusion matrix related to the predictions of nRC tool over the validation dataset (2600 sequences). We noticed the same behaviour as in the confusion matrix shown in Fig. 9, with a similar trend with regards to classification mistakes, highlighted in *red*

As mentioned before, to confirm there is not overfitting with regards to both feature extraction and the learning of the CNN, another confusion matrix (Fig. 10), has also been computed for the experiment with the independent validation dataset. Once again, we noticed the same behaviour as in the previous case, with a similar trend with regards to classification mistakes, such as the miRNA-snoRNA (CD-box and HACA-box) and CD-box-HACA-box misclassifications.

All these evidence let us hypothesise that all these classes of ncRNAs have some shared sub-features on the other analysed ncRNA classes. Because our approach considers these sub-structures as local features, the misclassification among some of the ncRNA classes could be explained by those shared features. Concluding, therefore, in spite of the overall good performances of our classification approach, we need to carry out some further analysis for the ncRNA classes whose sensitivity and a precision score was about 50%. A deeper investigation would allow us to increase the classification scores and to try understanding if and what are the relations between RNA sub-classes, considering, for example, global features as well.

## Conclusions

In this work, we introduce nRC (noncoding RNA Classifier), a new tool for the classification of non-coding RNA sequences. Three steps are the basis of the proposed method: the prediction of ncRNAs secondary structures, the extraction of frequent substructures as features and the classification of known ncRNA classes. To implement these processes, we used the IPknot algorithm to predict RNA secondary structures with pseudoknots, the MoSS decision tree pruning algorithm to obtain sub-structures, and a deep learning network architecture, namely a convolutional neural network, as a supervised classifier. Differently to other existing ncRNA classification approaches, we (i) created a ncRNAs vs. local topological features Boolean matrix as input data and (ii) adopted a DL architecture for classification. To demonstrate the effectiveness of the proposed approach, we first compared the proposed classifier with four of the most well-known classification algorithms, i.e. RF, NB, kNN and SVM, and then we compared our method with the RNAcon tool, that is the literature reference classifier of ncRNA sequences. Experiments have also been carried out using an independent validation dataset. In both tests, we demonstrated the advantages of using our approach on other strategies, obtaining the highest scores in terms of five different statistical measures, i.e. accuracy, sensitivity, specificity, precision, F-score and MCC. In particular, results demonstrated the proposed method outperformed the state-of-the-art RNAcon approach, doubling its performance in terms of accuracy and sensitivity.

As future work, we are working to train a classification model with much more ncRNA sequences, also belonging to some other well studied ncRNA classes, such as piwi-interacting RNA (piRNA) [53] and circular RNA (circRNA) [54]. In addition, to improve classification performances, we are planning to test some new secondary structure prediction tools, like those proposed in [55, 56]. Finally, we aim at creating a publicly available web service for the classification of unlabelled non-coding RNA sequences.

## Abbreviations

CNN: Convolutional neural network
DL: Deep learning
IRES: Internal ribosome entry site
kNN: K nearest neighbour
lncRNA: Long non-coding RNA
miRNA: MicroRNA
MoSS: Molecular sub-structure miner
NB: Naive Bayes
ncRNA: Non-coding RNA
nRC: Non-coding RNA classifier
RF: Random forest
rRNA: Ribosomal RNA
tRNA: Transfer RNA
siRNA: Silencing RNA
snoRNA: Small nucleolar RNA
snoRNP: Small nucleolar ribonucleoproteins
snRNA: Small nuclear RNA
SVM: Support vector machine
